# In vitro DNA repair genomics using XR-seq with *Escherichia coli* and mammalian cell-free extracts

**DOI:** 10.1073/pnas.2314233120

**Published:** 2023-10-16

**Authors:** Xuemei Cao, Cansu Kose, Christopher P. Selby, Aziz Sancar

**Affiliations:** ^a^Department of Biochemistry and Biophysics, University of North Carolina School of Medicine, Chapel Hill, NC 27599

**Keywords:** excision repair sequencing, transcription-coupled repair, chromatin, DNA damage

## Abstract

The XR-seq (eXcision Repair-sequencing) method has been extensively used to map nucleotide excision repair genome-wide in organisms ranging from *Escherichia coli* to yeast, *Drosophila*, Arabidopsis, mice, and humans. The basic feature of the method is to capture the excised oligomers carrying DNA damage, sequence them, and align their sequences to the genome. We wished to perform XR-seq in vitro with cell-free extract supplemented with a damaged DNA substrate so as to have greater flexibility in investigating factors that affect nucleotide excision repair in the cellular context [M. J. Smerdon, J. J. Wyrick, S. Delaney, *J. Biol. Chem.* **299**, 105118 (2023)]. We report here the successful use of ultraviolet light-irradiated plasmids as substrates for repair in vitro and in vivo by *E. coli* and *E. coli* cell-free extracts and by mammalian cell-free extract. XR-seq analyses demonstrated common excision product length and sequence characteristics in vitro and in vivo for both the bacterial and mammalian systems. This approach is expected to help understand the effects of epigenetics and other cellular factors and conditions on DNA repair.

## Significance

DNA damage caused by UV light and chemical agents that produce bulky adducts is removed in the form of damage-containing oligonucleotides 12–13 (prokaryotes) and 26–27 (eukaryotes) bases in length by nucleotide excision repair. XR-seq (eXcision Repair-sequencing) is a method developed to capture the excised oligonucleotides from cells or tissues and sequence and align the excision products to the genome at the single-nucleotide level. Here, we describe in vitro methods for XR-seq in both *Escherichia coli* and mammalian cells, using cell-free extracts and ultraviolet light-damaged plasmids. Comparison of in vitro with in vivo XR-seq repair maps are expected to provide novel insights on the effects of DNA higher order structure and histone modifications and chromatinization on DNA damage formation and repair.

In recent years, numerous genomic analysis methods have been developed to understand the various factors that affect the structure and function of the genome in variety of organisms (1, 2). To investigate, genome-wide, nucleotide excision repair in vivo and the effects of variables such as damage type, sequence context, chromatin state, transcription, replication, and the circadian clock on excision repair, we developed the XR-seq (eXcision Repair-sequencing) method (3, 4). In XR-seq technology, the excised DNA fragments carrying the damage (12–13-nt-long in prokaryotes and 26–27-nt-long in eukaryotes) are captured by immunoprecipitation with damage-specific antibodies that bind to proteins that are associated with the excised oligonucleotide (anti-TFIIH or anti-XPG antibodies in mammalian cells). The isolated oligonucleotides are then sequenced by next-generation sequencing and aligned to the genome. XR-seq and its later iterations have provided valuable information on factors that affect excision repair in organisms ranging from *Escherichia coli* to humans and provided genome-wide repair maps of DNA damage induced by ultraviolet light (UV), cisplatin, bezo[a]pyrene, and other carcinogenic or therapeutic DNA damaging agents (5–11). Even though with this approach valuable data were obtained, the options for testing the effects of the many cellular variables that affect the chromatin and DNA states on excision repair in eukaryotes and prokaryotes are limited. Thus, we wished to develop an XR-seq system in vitro where the reaction conditions can be readily controlled and use this system for genome-wide analysis of excision repair under desired reaction conditions for comparison with in vivo data. To this end, as a first step, we decided to use *E. coli* and mammalian cell-free extracts (CFE) that are known to be proficient in excision repair, and a UV-damaged plasmid carrying both a prokaryotic gene and promoter and a eukaryotic gene and promoter for in vitro XR-seq. We found that the excision repair patterns produced using CFE were in agreement with the published in vivo XR-seq data. However, even though the mammalian CFE did not perform transcription-coupled repair (TCR) because of very low transcription efficiency in the in vitro assay, the *E. coli* CFE performed TCR in vitro comparable in magnitude to the in vivo TCR. We expect that with further refinement, in vitro XR-seq will be applied to the entire prokaryotic and eukaryotic genomes and it will provide information complementary to in vivo XR-seq.

## Results and Discussion

**Fig. 1.**
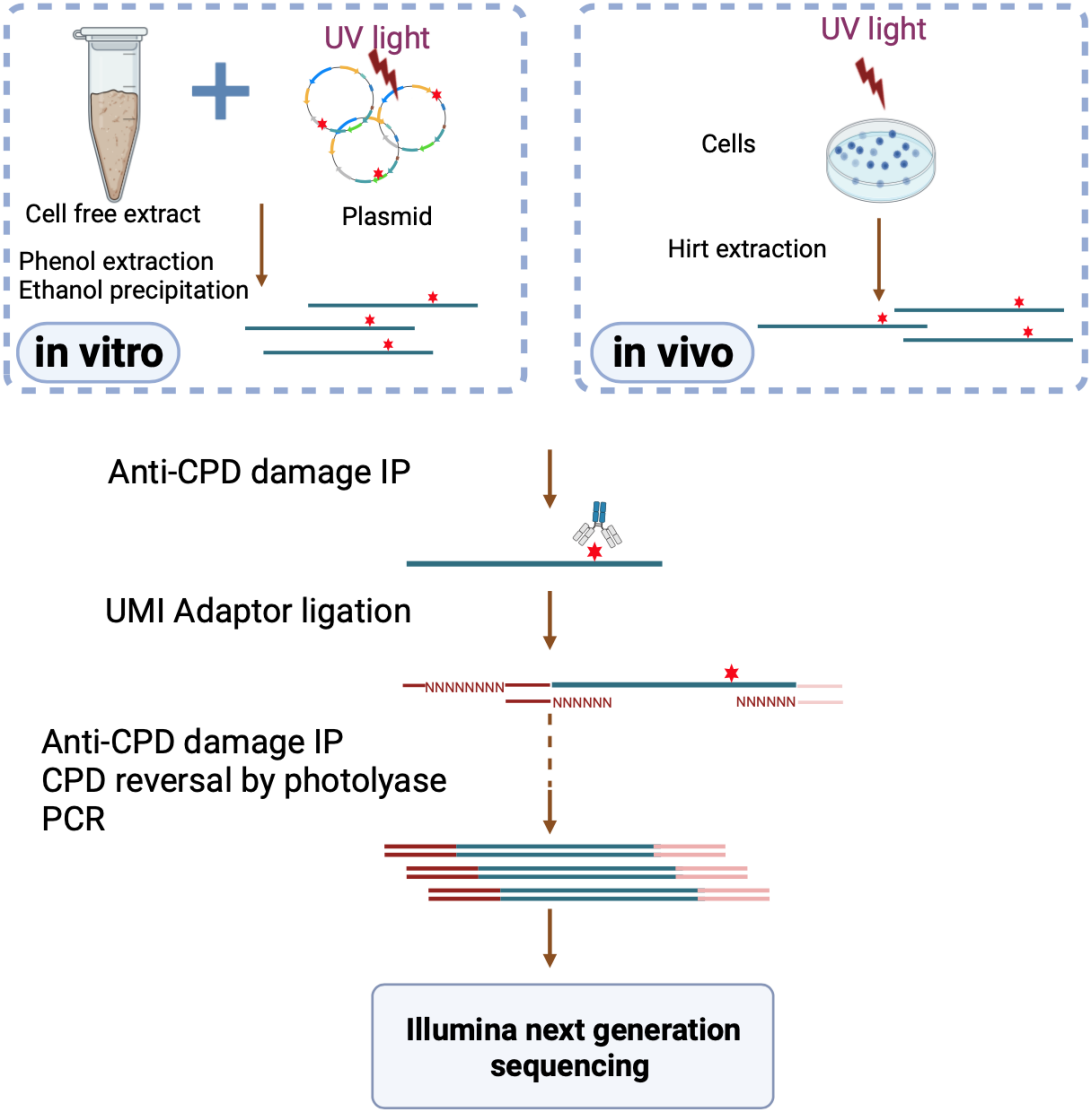
Schematic representation of in vivo and in vitro XR-seq. Red stars indicate UV-induced cyclobutane pyrimidine dimer (CPD) damage in DNA. In in vitro XR-seq (*Left*), ultraviolet light (UV)-irradiated plasmid is incubated with CHO or *E. coli* CFE, and excision products are isolated by phenol extraction and ethanol precipitation. In in vivo XR-seq (*Right*), after damage induction, at selected repair times, cells are collected and subjected to Hirt lysis. Then, the excised oligonucleotides (blue) are purified with anti CPD antibodies. Next, adapters, one of which has an 8-nt random, unique molecular identifier (UMI) sequence, are ligated and then a second anti-CPD purification is done. Finally, CPDs are repaired by photoreactivation to allow PCR generation of a sequencing library. The relatively small plasmid DNA substrate allows relatively complete coverage of repair sites. Use of the unique molecular identifier sequence allows multiple reads generated from any single site to be categorized as genuine multiple reads or artefactual, multiple reads generated by PCR. The latter are removed in the data analysis.

In order to evaluate the contributions of epigenetic factors such as DNA and chromatin modifications and the 3-D structure of chromatin to excision repair (12), we wished to perform XR-seq in vitro using *E. coli* and mammalian CFE and compare the outcome of these in vitro repair reactions with the in vivo repair reactions on the same genes. The overall strategy is presented in Fig. 1. The substrate is a plasmid carrying a gene (*bla* = beta lactamase which confers penicillin/ampicillin resistance to *E. coli*) under an *E. coli* RNA Polymerase (RNAP) promoter and a gene (*mPer1*= mouse *Period1* gene which controls the circadian clock) under an RNAPII promoter (*SI Appendix*, Fig. S1). The aim was to observe the repair of these genes in this in vitro repair system and the effect of transcription on these genes for comparison with the in vivo effect observed for both genes in their respective prokaryotic and eukaryotic hosts (13, 14).

### Excision Assay.

**Fig. 2.**
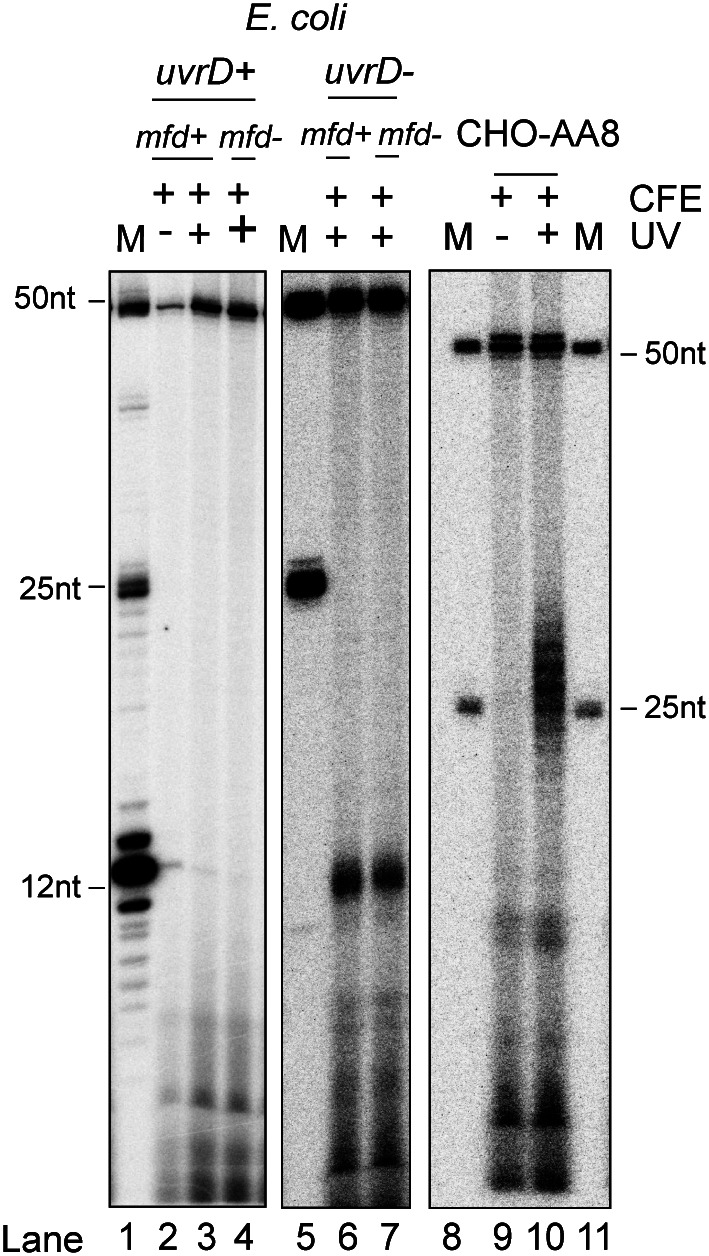
Excision products generated with *E. coli* CFE are protected from degradation in uvrD^−^ cells. Excision products generated from UV-irradiated plasmid upon incubation with *E. coli uvrD*^+^ CFE are shown in the left panel for comparison. The middle panel shows levels of excision following incubation of the plasmid with *uvrD*^−^ extracts. Deletion of *mfd* had no effect on overall excision, as reported previously (6, 18). 50-nt, 25-nt and 12-nt size markers are in lanes M. The right panel shows excision levels obtained using CHO-AA8 CFE.

XR-seq studies to date have analyzed excision products harvested from cells or tissues. To begin our studies, we performed excision assays to determine whether we could generate and isolate excision products following incubation of an UV-irradiated plasmid with CFE. In the excision assay, the UV-irradiated plasmid is incubated with the appropriate CFE for 5 min for *E. coli* and for 60 min for Chinese Hamster Ovary (CHO) CFE, and then, the excised DNA is labelled with alpha-^32^P deoxyadenosine 5′ triphosphate using terminal transferase, and the products are separated on a sequencing gel (6, 15). Fig. 2 shows the result of such an experiment. Some noteworthy points from this figure are the following. First, with *E. coli* wild type with respect to UvrD helicase (Fig. 2 lanes 2 to 4), the excised oligonucleotide 12–13nt in length typical of prokaryotes (16) is marginally or not detected at all whether cells carry wild-type or mutated transcription-repair coupling factor Mfd. This is in agreement with previous in vivo observations that in wild-type *E. coli* following the dual incision, 12–13 nucleotides apart, by the Uvr(A)BC excision nuclease, the excised fragment along with the uvrB and uvrC subunits are displaced from the excision gap by UvrD helicase (17) and the released 12–13nt-long oligomers are rapidly degraded by multiple exonucleases present in *E. coli* (6, 14). Thus, large numbers of wild-type *E. coli* cells are needed to isolate sufficient quantities of excised oligonucleotide for genomic processing. Since our goal was to isolate sufficient quantities of excised oligomer and to determine the effect of Mfd, the transcription repair coupling factor (TCRF), on the distribution of the excised oligomers in the transcribed strand vs. the nontranscribed strand, we used *uvrD* mutants with or without the *mfd* mutation in our excision assay (Fig. 2 lanes 6 to 7). As was demonstrated previously, in the *uvrD^−^* background, the full-size 12–13 mers are obtained in good yield in both the *mfd^+^* and *mfd^−^* backgrounds (6). Thus, we used the excised oligonucleotides from this and replica batches for genomic analysis.

With regard to mammalian cells, the excised oligonucleotides are known to be released from the excision gap in a complex with TFIIH and XPG (3, 18, 19) and, thus, protected from nonspecific nuclease attack for 1 h or longer, and hence in wild-type background [in particular in CHO cells which are relatively slow in excised oligonucleotide degradation (20)], the excised oligomers in the range of 24 to 28 nucleotides are readily detectable 1 h after the excision reaction (Fig. 2 lane 10).

### Analysis of In Vitro and In Vivo Excision Products (XR-Seq) for *E. coli* and Mammalian Cells.

**Fig. 3.**
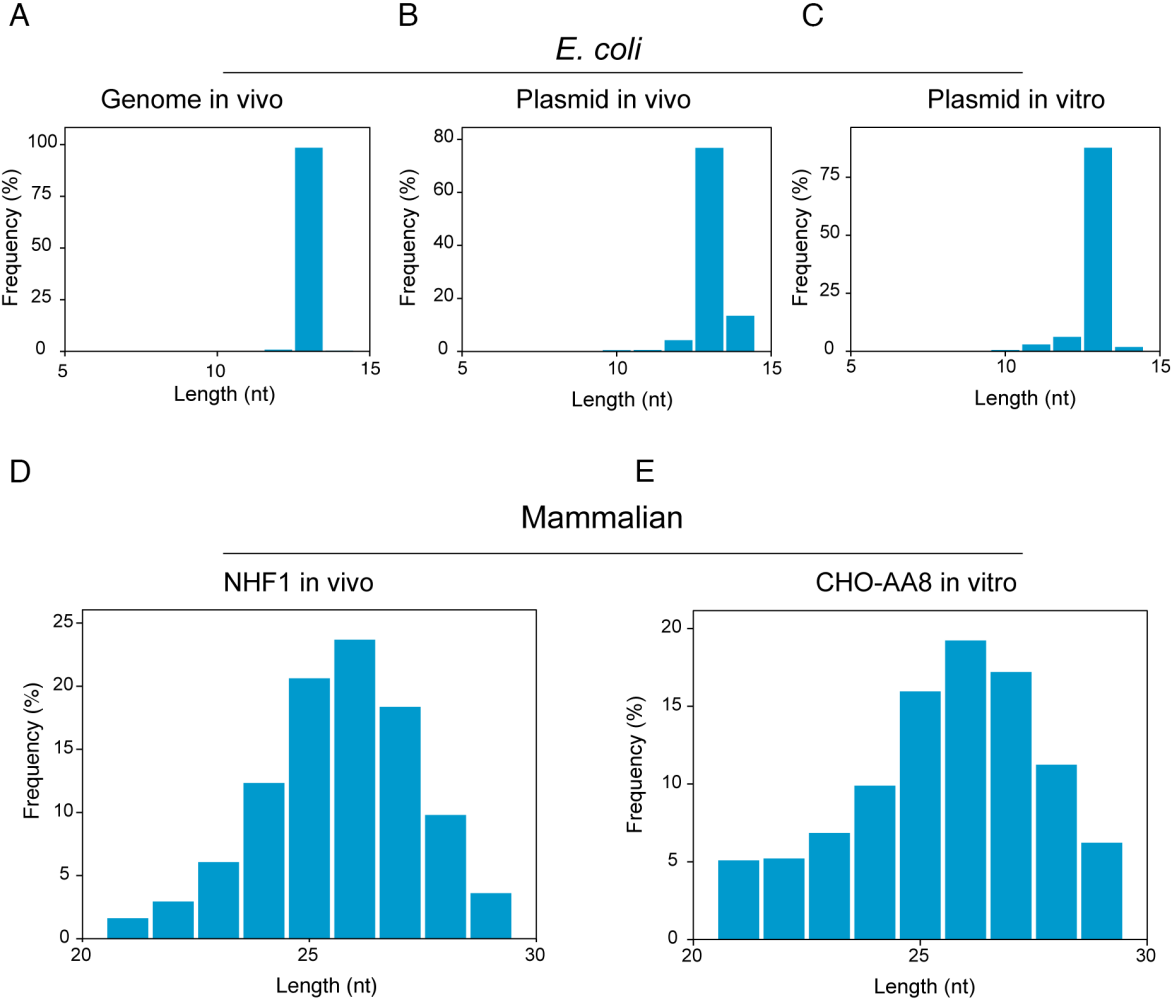
Length (in nt) of excised oligonucleotides from *E. coli* and mammalian cells and extracts as determined by XR-seq. (*A*–*C*) *E. coli* cells and extract. (*D*) is from NHF1 cells irradiated in vivo, from ref. 3, and (*E*) is from CHO AA8 cell-free extract.

**Fig. 4.**
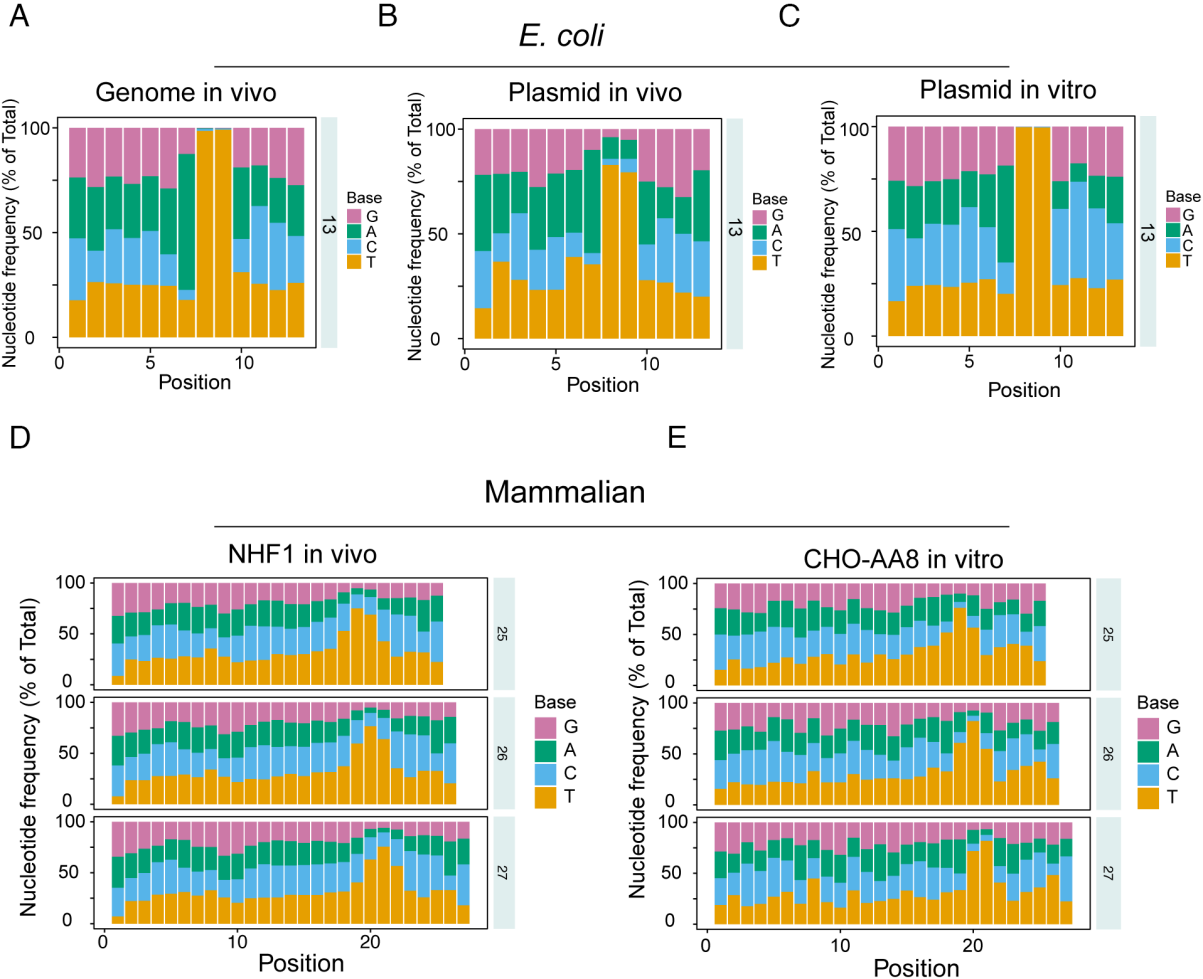
Distribution of nucleotides within excision products generated in vitro and in vivo in *E. coli* (*A*–*C*) and in mammalian cells (*D* and *E*) as determined by XR-seq. Note that in *E. coli*, oligonucleotides shorter than 12-mer are nucleolytic degradation products while those above 13 nt constitute a very small fraction of the total excised oligomers (see Fig. 2), while in mammalian cells even though the peak is at 26–27 nts, those in the range of 26 to 30 are all primary products as they have also been obtained from excision assays with a highly purified human 6-factor excision nuclease system (18, 21). The 5′ end of excision products is located at position 1.

Our ability to obtain large quantities of excision products from the in vitro reactions provided the opportunity to compare the in vivo and in vitro reactions with regard to size distribution, excised oligonucleotide sequence pattern, and preferential repair sites of *E. coli* vs. CHO cells. Fig. 3 *A–C* show that for the *E. coli* genome and the plasmid repaired in vivo or in vitro, by far the major product is 13nt in length. However, some minor variations are seen with the plasmid repair fragment size with about 10% 14 mers in the in vivo repaired plasmid and about 5% 12-mer in the in vitro repaired plasmid. Because of the relatively small fractions of these outlier sizes, we did not investigate in detail whether noncanonical sizes are real or an artifact of relatively high concentration of plasmid relative to the genomic DNA. Fig. 3 *D* and *E* show that, as in *E. coli,* in mammalian cells also the CFE excision pattern in vitro essentially imitates the excision pattern seen with the in vivo reaction (3), with fragments 25 to 27 with T-T dimer at 5 to 6 nucleotides from the 3′ end dominating (Fig. 4 *D* and *E*). The minor differences seen between the in vitro and in vivo size distribution are of the magnitude seen from one experiment to another under in vivo conditions. Fig. 4 *A*–*C* show nucleotide composition of the excised fragments from the *E. coli* genome in vivo, the plasmid in vivo, and the plasmid in the in vitro reaction. The in vivo genome and in vitro plasmid excision products contain T-T at the 8 to 9 positions essentially in 100% of the 13-mers while in vivo plasmid excision products contain the other three bases in a small fraction of the 8 to 9 positions, some of which might be real but were not pursued because of the relatively low numbers of these species. More interestingly, it appears that 5′ to the T-T dimer the base residue A is preferred, reflecting selectivity in damage recognition or nucleotide distribution in *E. coli*. The percentage of A residues at the position 5′ to the dipyrimidine target sequence is 47% (in vivo plasmid), 45% (in vitro plasmid), and 63% (genome, in vivo) (Fig. 4 *A*–*C*), while the percentage of A residues located 5′ to TT dinucleotides is 22% in the plasmid and 25% in the *E. coli* genome (*SI Appendix*, Fig. S2).

### Sequence Effect on Repair in Mammalian Cells and *E. coli*.

**Fig. 5.**
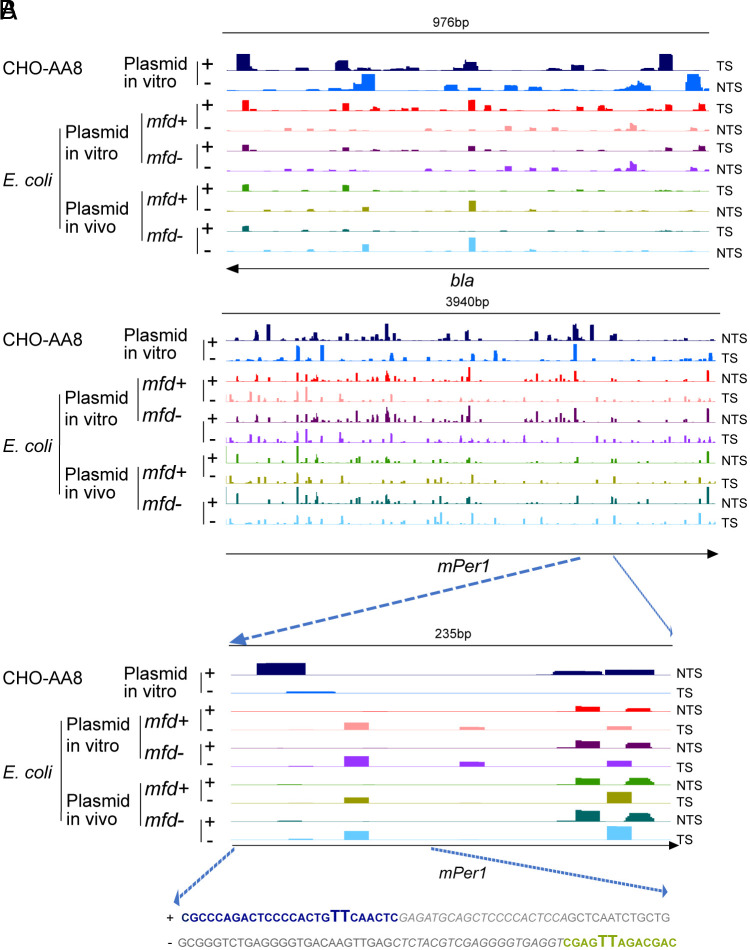
XR-seq analysis of plasmid repair by *E. coli* cells in vivo, and by *E. coli* and CHO-AA8 CFE in vitro. Panel *A* shows a representative browser view (screenshot) of in vitro CPD repair signals (*Y* axis) in the plasmid-borne *bla* gene (*X* axis). Repair of both strands (+ and −) is shown; the + strand is the transcribed strand, and enhanced repair is seen in the transcribed strand repaired by *mfd*^+^ cell extract as compared to repair by the *mfd*^−^ cell extract. The plasmid is repaired but not transcribed by the CHO-AA8 extract. Panel *B* shows repair by *E. coli* and CHO extracts of 3,940 bp of the *mPer1* coding sequence, and the panel below zooms in on a 235 bp region. Further zooming in to a 60 nt region, at the bottom, emphasizes that in some cases different excision products are preferentially generated from the same UV-irradiated plasmid by the different species. In this case, the CHO extract excises a fragment (blue) with a CPD (TT in bold) in one strand while the *E. coli* extract excises a fragment (green) with a CPD (TT in bold) in the other strand. In contrast to the results in panel *A* showing TCR of *bla*, the *mPer1* coding sequence is not appreciably transcribed by either *E. coli* or CHO extracts and no TCR of *mPer1* is detected. (The—strand is nominally the TS for *mPer1* transcription from the mammalian promoter.)

There have been suggestions that even though both *E. coli* and mammalian cells remove damage by dual incision, nucleotide excision repair in mammals and prokaryotes have different sequence preferences (22, 23) because the two excision repair systems are evolutionarily not related (24, 25). The availability of in vivo and in vitro XR-seq enabled us to observe this phenomenon directly. Fig. 5 *A* and *B* show screenshots of repair patterns of *bla* and *mPer1* genes in vitro and of *bla* gene both in vitro and in vivo in *mfd^+^* and *mfd^−^* backgrounds. As apparent, the repair patterns of both *bla* and *mPer1* are rather different between repair by CHO extract on the one hand and by the *E. coli* CFE or *E. coli* cells in vivo, providing direct evidence for the relative sequence preference of repair of the two systems. In Fig. 5*B*, the bottom panel shows that in a 235 bp region of the *mPer1* gene, the CPD repair pattern in the *E. coli* system under four different conditions (plasmid *mfd^+^*, plasmid *mfd^−^* in vitro and in vivo) is relatively well conserved, because this nontranscribed gene is not qualitatively affected by the presence or absence of Mfd. In contrast, the excision pattern for the same region in CHO-AA8 extract shows a rather different pattern. An example of two hotspots of repair unique to either CHO extract or *E. coli* extract is shown in the bottom of the figure in which the 26-mer of CHO repair and the 13-mer of *E. coli* repair are highlighted.

### TCR in CFE Analyzed by XR-Seq.

Repair of *mPer1* under the control of a RNAPII promoter in CFE of wild-type CHO-AA8 supplemented with or without rNTP failed to show preferential repair of the transcribed strand (*SI Appendix*, Table S1), in agreement with a previous observation showing very low RNAPII transcription and therefore TCR in the cell-free mammalian extract system developed to define the essential components of RNAPII transcription (26, 27). Therefore, we decided to focus on TCR by *E. coli* CFE using a plasmid substrate and compared the results of the TCR value of the *bla* gene in vitro and in vivo. In addition, we measured the repair in the *E. coli* genome of two noninducible but highly expressed genes (*pnp*, *rpoB*) and one inducible gene (*lacZ*) as internal references for TCR. Results are summarized in Table 1 (additional time points for *E. coli* in vitro repair are in *SI Appendix*, Table S2). The following points are notable. First, the poorly transcribed *bla* gene exhibits modest TCR (which is defined as *mfd*^+^ TS/NTS ratio/*mfd*^−^ TS/NTS ratio > 1) of 1.69 in vivo and 2.47 in vitro confirming several previous studies that *E. coli* CFE performs TCR (13, 14, 28). The higher value of in vitro TCR than in vivo TCR is presumably due to the fact that in vivo, Mfd, in addition to the plasmid, is engaged in TCR of the cellular genome and hence there is less Mfd to aid in the repair of the plasmid. Second, the highly expressed genes *pnp* and *rpoB* exhibit high values of TCR ~6. Third, the *lacZ* gene, which is commonly used for assaying TCR (29–31), shows no TCR (0.96) when cells are grown in glucose, and very high, Mfd-dependent TCR (6.2) upon induction with IPTG. Finally, the *mPer1* gene, which is carried on the same plasmid as *bla* but is under RNAPII promoter control shows no strand preference of repair either in vitro or in vivo and either in *mfd*^+^ or *mfd*^−^ background (TCR~1.0). In contrast, the highly homologous *hPer1* gene (88% sequence identity), when expressed on a plasmid from the tac promoter (+IPTG), did demonstrate Mfd-dependent TCR (Table 1). We note that this circadian clock gene exhibits TCR TS/NTS repair ratios ~50 in mouse liver cells at the peak of *Per1* oscillation (10, 32) and even TCR of ~20 in mouse tissue culture (11) which is asynchronous with regard to circadian rhythm across the tissue culture cell population.

**Table 1.** TCR in vitro and in vivo by *E. coli* CFE

		In vitro			In vivo		
		TS/NTS		TCR (*mfd*^+^*/mfd*^−^)	TS/NTS		TCR (*mfd*^+^*/mfd*^−^)
		*mfd* ^+^	*mfd* ^−^		*mfd* ^+^	*mfd* ^−^	
Plasmid	*bla*	1.63	0.66	2.47	0.83	0.49	1.69
	*mPer1* *(hPer1)*	0.84	0.77	1.09	0.89 (2.58)	0.89 (0.65)	1 (3.97)
*E. coli* genome	*rpoB*	NA	NA	NA	6.65	1.00	6.65
	*pnp*	NA	NA	NA	6.31	1.02	6.19
	*lacZ* (*+IPTG*)	NA	NA	NA	1.09 (5.54)	1.13 (0.89)	0.96 (6.20)

Values are given for the ratio of transcribed/nontranscribed strand (TS/NTS) repair in *mfd*^+^ and *mfd*^−^ cells. Values are also given for TCR, as the ratio of (TS/NTS) in *mfd*^+^ cells divided by the (TS/NTS) in *mfd*^−^ cells (*mfd*^+^/*mfd*^−^). The ratio of TT residues (potential sites for CPD formation) in the TS/NTS for *bla* is 0.96, and for *mPer1,* it is 1.15. Repair values were not normalized to TT frequencies. For *lacZ*, values in parentheses were obtained from cells without plasmid incubated with IPTG. The values in parentheses for *hPer1* are for plasmid-borne *Per1* under the *lacZ* promoter/operator control, +IPTG, in vivo. NA, not applicable.

## Conclusion

After the discovery of TCR, first in mammalian cells and subsequently in *E. coli* cells (29, 33) using the traditional T4 endonuclease V assay, in vitro methods to measure excision repair in the form of incision and repair synthesis assays were developed for identification and characterization of Mfd (TCRF) in *E. coli* (13, 28). More recently, the in vivo XR-seq method was developed to study in vivo TCR both in mammalian cells and in *E. coli* (3, 6). Here, we have adopted the XR-seq assay to analyze TCR at the genomic level both in *E. coli* and in mammalian cells. The XR-seq in vitro results obtained with *E. coli* have given results consistent with those obtained by the incision (13, 28) and repair synthesis assay (13) but in much greater detail and at the genomic scale. The in vitro XR-seq results for mammalian cells have been useful in confirming and extending the in vivo XR-seq results with regard to the overall operation of mammalian excision repair and its difference in terms of sequence preference from *E. coli* excision repair. However, in contrast to the *E. coli* in vitro XR-seq which reveals both TCR and repair of nontranscribed DNA (global repair), the mammalian in vitro XR-seq so far has not been conducive to detection of TCR because of the well-known very limited transcription in the mammalian in vitro RNAPII transcription systems and waits more proficient systems. Nevertheless, Genomic XR-seq in vitro has the potential of identifying repair accessible genomic regions in a manner similar to ATAC-seq (34) and eventually achieving TCR of mammalian cells in vitro that has not been possible with naked DNA so far.

## Materials and Methods

### Plasmids, Strains, and Extracts.

Plasmids pcDNA3.1/V5-His-*mPer1* (35) and pGST-*hPer1* were irradiated with 225 J/m^2^ UVC for this study. The former plasmid contains 3,873 bp of the *mPer1* coding sequence positioned for expression from the cytomegalovirus enhancer/promoter in transfected mammalian cells, as well as the *bla* gene for selection in bacteria (*SI Appendix*, Fig. S1*A*). The latter plasmid contains 4021 bp of the *hPer1* coding sequence in-frame downstream from the *GST* gene in pGEX3x, in which the *GST* transcriptional unit is under control of the *tac* promoter (*SI Appendix*, Fig. S1*B*).

*E. coli* strains MPdD and MPdDdM (7) were used for making extracts. MPdD contains deletions of the *phr* and *uvrD* genes. MPdDdM contains deletions of the *phr*, *uvrD*, and *mfd* genes and was generated by generalized transduction of MPdDd with phage P1 using strain JW1100-1 [mfd-739(del)::kan] from the Keio collection (provided by the Coli Genetic Stock Center at Yale University) as a source of the *mfd* deletion. MGP (*phr*^−^) and MPdM (*phr*^−^, *mfd*^−^), also used for making extracts, and MPdDd have been described (7).

*E. coli* CFE were prepared as described originally by Wickner (36) with modifications of Lu et al. (37). Protein concentrations ranged from 43 to 80 mg/mL. Extracts of AA8-CHO cells (15 mg/mL) were prepared by the method of Manley (26).

### In Vitro Excision and XR-Seq Assays.

In vitro assays with *E. coli* extracts (1.2 mg/mL final) were conducted with 1.3 nM irradiated pcDNA3.1/V5-His-*mPer1* or pGST-*hPer1* substrates, 40 mM HEPES-KOH (pH 7.9), 50 mM KCl, 8 mM MgCl_2_, 100 µg/mL bovine serum albumin, 6% PEG, and 2 mM adenosine triphosphate, 200 µM CTP, 200 µM GTP, 200 µM UTP, 5 mM dithiothreitol, and 40 µM dNTPs. Reactions were done in a total 25 µL volume at 37 °C for 5, 12, or 25 min as described (38), in the absence of IPTG. The plasmid repair time for data in Table1 was 5 min. Excision assays utilized four reactions and XR-seq assays utilized 16 reactions.

Assays with mammalian extracts AA8 CFE (75 µg) were conducted with 200 ng of irradiated pcDNA3.1/V5-His-*mPer1* substrate, 30 mM HEPES-KOH (pH 7.9), 40 mM KCl, 3 mM MgCl_2_, 100 µg/mL bovine serum albumin, 6% PEG, and 4 mM adenosine triphosphate, 200 µM CTP, 200 µM GTP, 200 µM UTP, 5 mM dithiothreitol, 5 µg creatine kinase, 10 mM creatine phosphate, and 4 µg ubiquitin with a total volume of 25 µL at 30 °C for 60 min as described (20). One reaction was used per excision assay and 10 reactions were used per XR-seq assay.

For both prokaryotic and eukaryotic assays, after the excision reaction, DNA was extracted with phenol:chloroform:isoamyl alcohol and precipitated with ethanol, and resuspended in immunoprecipitation reaction buffer. The excision products were then isolated from DNA by IP with anti-CPD antibody. For excision assays, excision products were then radiolabeled with ^32^P and separated with a 12% (mammalian) or 16 % (*E. coli*) sequencing gel.

For XR-seq assay, instead of radiolabeling samples, DNA was ligated to adapters designed for amplification for next-generation sequencing. The adapters for mammalian cells (4) and prokaryotic cells (7) have been described. Since the bacterial genome and the plasmids are relatively small, multiple excision product reads per potential damage site were possible. Thus, to enable identification and disposal of artefactual multiple reads generated by PCR, the bacterial adapters were designed to include an 8-nucleotide unique molecular identifier sequence. Following ligation of adapters, samples were immunoprecipitated with anti-CPD antibody to purify the ligated products from free adapters and oligonucleotides. Samples were then processed for sequencing (XR-Seq) as previously described (4, 6, 7). All in vivo and in vitro excision assays and XR-seq experiments were performed at least two times and representative results are shown.

### In Vivo Excision and XR-Seq Assays.

Assays with mammalian cells (3, 4) and *E. coli* were done as described previously (6, 7). In this study, for in vivo XR-seq assays with *E. coli*, transformed or nontransformed MPdD or MPdDdM saturated overnight cultures were diluted in LB 1/50 to start cultures for experiments. IPTG, when included, was added at 1 mM when cultures were started (no plasmid) or when cultures reached an OD of approximately 0.42 or 10 min before harvesting (plasmid-transformed cells). Glucose was added to 0.4% (v/v) when included. When cultures reached an OD_600_ of approximately 0.5, they were brought from 37° to room temperature with agitation in a cold water bath, and then cells were transferred to four to six R-150 dishes (15 mL per dish). Cells were irradiated with agitation with 100 J/m^2^ UVC (principally 254 nm, at 2 J/m^2^/sec) and incubated at room temperature for repair. Repair was stopped after 5 min repair by placing each dish on ice water. In one exception, repair of *LacZ*+IPTG in Table1, repair was for 1 min. Samples were processed for XR-seq as described above.

### Statistical and Data Analyses.

The in vivo NHF1 CPD XR-seq raw data were obtained from Hu et al. (3). and are available on the Gene Expression Omnibus (GEO), accession number GSE67941. Adaptor sequences were trimmed by cutadapt (39), and duplicate reads were removed by fastx_toolkit/0.0.14 (hannonlab.cshl.edu/fastx_toolkit/index.html). Then, the reads were aligned to hg38_UCSC reference genome by bowtie2 (40) with -f -very-sensitive arguments. For in vivo *E. coli* data analysis, after removing duplicate reads, adaptor sequence with the unique molecular identifier were trimmed by cutadapt. The reads were aligned to the *E. coli* reference genome retrieved from the National Center for Biotechnology Information (NCBI, accession number NC_000913.2) using bowtie2 with -f -very-sensitive arguments. For in vitro XR-seq data, duplicate reads were removed and adaptor with the unique molecular identifier were trimmed by cutadapt. To generate reference genome fasta and bed files, the plasmid, pcDNA3.1/V5-His-*mPer1*, which was subjected to the repair by either *E. coli* or CHO cell-free extract, was sequenced. The reads were aligned by BWA-backtrack (41), followed by Picard tools (https://broadinstitute.github.io/picard/) for filtering, sorting, deduplication, and indexing. Bamtools (42) was used to remove reads with any mismatches/gaps.

Oligonucleotide lengths and nucleotide distributions were plotted by R. For data visualization, only 26-nt in length with dipyrimidine at 19th to 20th positions XR-seq reads with CHO cell-free extract and only 13 nt in length with dipyrimidine at eighth to ninth positions XR-seq reads with *E. coli* cell-free extract were selected, then subsampled to 200,000 reads. The bigwig file is visualized by integrated genomic viewer (software.broadinstitute.org/software/igv/, Broad Institute, and the Regents of the University of California) (43).

## Supplementary Material

Appendix 01 (PDF)

## Data Availability

The raw data have been deposited in the Sequence Read Archive (SRA) of the National Center for Biotechnology Information (NCBI) under accession number PRJNA1004315 (44).
