# Supplementary material for: In vitro DNA repair genomics using XR-seq with Escherichia coli and mammalian cell-free extracts
Source: Proc Natl Acad Sci U S A. 2023 Oct 16;120(43):e2314233120. doi: 10.1073/pnas.2314233120 (PMC10614213; doi:10.1073/pnas.2314233120)
Supplement: Supplementary file 1 — Appendix 01 (PDF) [file pnas.2314233120.sapp.pdf]

PNAS
www.pnas.org

Supplementary Information for

In vitro DNA Repair Genomic Using XR-seq with *Eshcerichia coli* and Mammalian Cell-Free Extracts

Xuemei Cao[1], Cansu Kose[1], Christopher P. Selby, and Aziz Sancar[2]

Department of Biochemistry and Biophysics
University of North Carolina School of Medicine
Chapel Hill, North Carolina 27599

[1] Equal contribution
[2] Correspondence: aziz_sancar@med.unc.edu

This PDF file includes:
Figure S1 and S2
Tables S1 and S2

Figure S1 pcDNA3.1/V5-His-*mPer1* **map and sequence**

A, pcDNA3.1/V5-His-*mPer1*-Map

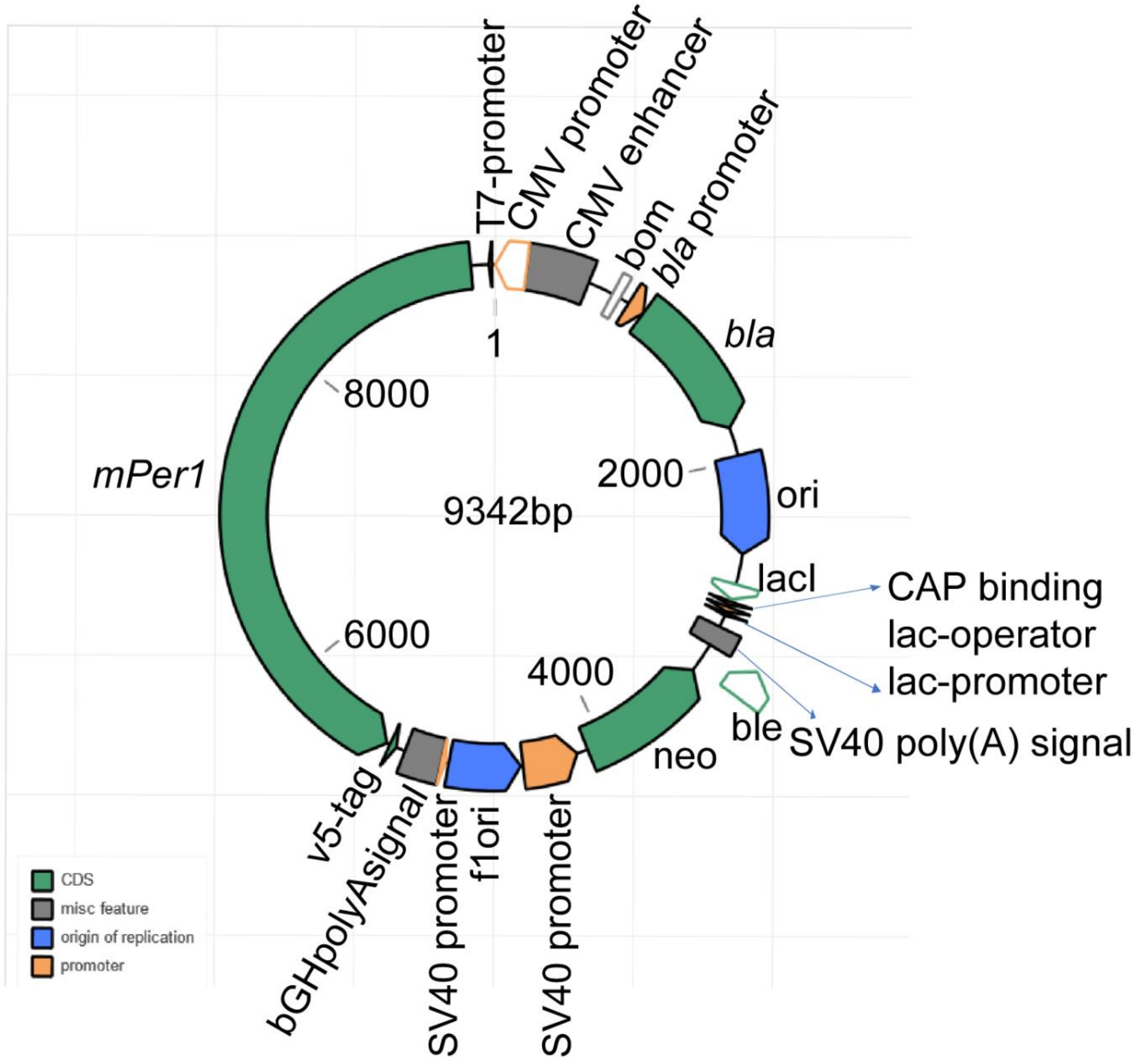

B, pcDNA3.1/V5-His-*mPer1* sequence

>mPer1
GACCTCCCACCGTACACGCCTACCGCCCATTTGCGTCAATGGGGCGGAGTTGTTACGACATTTTGGAAAGTCCCGTTGATTTTGGTG
CCAAAACAAACTCCCATTGACGTCAATGGGGTGGAGACTTGGAAATCCCCGTGAGTCAAACCGCTATCCACGCCCATTGATGTACT
GCCAAAACCGCATCACCATGGTAATAGCGATGACTAATACGTAGATGTACTGCCAAGTAGGAAAGTCCCATAAGGTCATGTACTGGG
CATAATGCCAGGCGGGCCATTTACCGTCATTGACGTCAATAGGGGGCGTACTTGGCATATGATACACTTGATGTACTGCCAAGTGGGC
AGTTTACCGTAAATACTCCACCCATTGACGTCAATGGAAAGTCCCTATTGGCGTTACTATGGGAACATACGTCATTATTGACGTCAAT
GGGCGGGGGTCGTTGGGCGGTCAGCCAGGCGGGCCATTTACCGTAAGTTATGTAACGCGGAACTCCATATATGGGCTATGAACTAAT
GACCCCGTAATTGATTACTATTAATAACTAGTCAATAATCAATGTCAACGCGTATATCTGGCCCGTACATCGCGAAGCAGCGCAAAAC
GCCTAACCCTAAGCAGATTCTTCATGCAATTGTCGGTCAAGCCTTGCCTTGTTGTAGCTTAAATTTTGCTCGCGCACTACTCAGCGAC
CTCCAACACACAAGCAGGGAGCAGATACTGGCTTAACTATGCGGCATCAGAGCAGATTGTACTGAGAGTGCACCATAGGGGATCGG
GAGATCTCCCGATCCGTCGACGTCAGGTGGCACTTTTCGGGGAAATGTGCGCGGAACCCCTATTTGTTTATTTTTCTAAATACATTCA
AATATGTATCCGCTCATGAGACAATAACCCTGATAAATGCTTCAATAATATTGAAAAAGGAAGAGTATGAGTATTCAACATTTCCGTG
TCGCCCTTATTCCCTTTTTTGCGGCATTTTGCCTTCCTGTTTTTGCTCACCCAGAAACGCTGGTGAAAGTAAAAGATGCTGAAGATCA
GTTGGGTGCACGAGTGGGTTACATCGAACTGGATCTCAACAGCGGTAAGATCCTTGAGAGTTTTCGCCCCGAAGAACGTTTTCCAA
TGATGAGCACTTTTAAAGTTCTGCTATGTGGCGCGGTATTATCCCGTATTGACGCCGGGCAAGAGCAACTCGGTCGCCGCATACACT
ATTCTCAGAATGACTTGGTTGAGTACTCACCAGTCACAGAAAAGCATCTTACGGATGGCATGACAGTAAGAGAATTATGCAGTGCTG
CCATAACCATGAGTGATAACACTGCGGCCAACTTACTTCTGACAACGATCGGAGGACCGAAGGAGCTAACCGCTTTTTTGCACAAC
ATGGGGGATCATGTAACTCGCCTTGATCGTTGGGAACCGGAGCTGAATGAAGCCATACCAAACGACGAGCGTGACACCACGATGCC
TGTAGCAATGGCAACAACGTTGCGCAAACTATTAACTGGCGAACTACTTACTCTAGCTTCCCGGCAACAATTAATAGACTGGATGGA
GGCGGATAAAGTTGCAGGACCACTTCTGCGCTCGGCCCTTCCGGCTGGCTGGTTTATTGCTGATAAATCTGGAGCCGGTGAGCGTG
GGTCTCGCGGTATCATTGCAGCACTGGGGCCAGATGGTAAGCCCTCCCGTATCGTAGTTATCTACACGACGGGGAGTCAGGCAACTA
TGGATGAACGAAATAGACAGATCGCTGAGATAGGTGCCTCACTGATTAAGCATTGGTAACTGTCAGACCAAGTTTACTCATATATACT
TTAGATTGATTTAAAACTTCATTTTTAATTTAAAAGGATCTAGGTGAAGATCCTTTTTGATAATCTCATGACCAAAATCCCTTAACGTG
AGTTTTCGTTCCACTGAGCGTCAGACCCCGTAGAAAAGATCAAAGGATCTTCTTGAGATCCTTTTTTTCTGCGCGTAATCTGCTGCTT
GCAAACAAAAAAACCACCGCTACCAGCGGTGGTTTGTTTGCCGGATCAAGAGCTACCAACTCTTTTTCCGAAGGTAACTGGCTTCA
GCAGAGCGCAGATACCAAATACTGTTCTTCTAGTGTAGCCGTAGTTAGGCCACCACTTCAAGAACTCTGTAGCACCGCCTACATACC
TCGCTCTGCTAATCCTGTTACCAGTGGCTGCTGCCAGTGGCGATAAGTCGTGTCTTACCGGGTTGGACTCAAGACGATAGTTACCGG
ATAAGGCGCAGCGGTCGGGCTGAACGGGGGGTTCGTGCACACAGCCCAGCTTGGAGCGAACGACCTACACCGAACTGAGATACCT
ACAGCGTGAGCTATGAGAAAGCGCCACGCTTCCCGAAGGGAGAAAGGCGGACAGGTATCCGGTAAGCGGCAGGGTCGGAACAGG
AGAGCGCACGAGGGAGCTTCCAGGGGGAAACGCCTGGTATCTTTATAGTCCTGTCGGGTTTCGCCACCTCTGACTTGAGCGTCGAT
TTTTGTGATGCTCGTCAGGGGGGCGGAGCCTATGGAAAAACGCCAGCAACGCGGCCTTTTTACGGTTCCTGGCCTTTTGCTGGCCTT
TTGCTCACATGTTCTTTCCTGCGTTATCCCCTGATTCTGTGGATAACCGTATTACCGCCTTTGAGTGAGCTGATACCGCTCGCCGCAGC
CGAACGACCGAGCGCAGCGAGTCAGTGAGCGAGGAAGCGGAAGAGCGCCCAATACGCAAACCGCCTCTCCCCGCGCGTTGGCCG
ATTCATTAATGCAGCTGGCACGACAGGTTTCCCGACTGGAAAGCGGGCAGTGAGCGCAACGCAATTAATGTGAGTTAGCTCACTCA
TTAGGCACCCCAGGCTTTACACTTTATGCTTCCGGCTCGTATGTTGTGTGGAATTGTGAGCGGATAACAATTTCACACAGGAAACAG
CTATGACCATGATTACGCCAAGCTCTAGCTAGAGGTCGACGGTATACAGACATGATAAGATACATTGATGAGTTTGGACAAACCACA
ACTAGAATGCAGTGAAAAAAATGCTTTATTTGTGAAATTTGTGATGCTATTGCTTTATTTGTAACCATTATAAGCTGCAATAAACAAGT
TGGGGTGGGCGAAGAACTCCAGCATGAGATCCCCGCGCTGGAGGATCATCCAGCCGGCGTCCCGGAAAACGATTCCGAAGCCCAA
CCTTTCATAGAAGGCGGCGGTGGAATCGAAATCTCGTGATGGCAGGTTGGGCGTCGCTTGGTCGGTCATTTCGCGAACCCCAGAGT
CCCGCTCAGAAGAACTCGTCAAGAAGGCGATAGAAGGCGATGCGCTGCGAATCGGGAGCGGCGATACCGTAAAGCACGAGGAAG
CGGTCAGCCCATTCGCCGCCAAGCTCTTCAGCAATATCACGGGTAGCCAACGCTATGTCCTGATAGCGGTCCGCCACACCCAGCCGG
CCACAGTCGATGAATCCAGAAAAGCGGCCATTTTCCACCATGATATTCGGCAAGCAGGCATCGCCATGGGTCACGACGAGATCCTC
GCCGTCGGGCATGCGCGCCTTGAGCCTGGCGAACAGTTCGGCTGGCGCGAGCCCCTGATGCTCTTCGTCCAGATCATCCTGATCGA
CAAGACCGGCTTCCATCCGAGTACGTGCTCGCTCGATGCGATGTTTCGCTTGGTGGTCGAATGGGCAGGTAGCCGGATCAAGCGTAT
GCAGCCGCCGCATTGCATCAGCCATGATGGATACTTTCTCGGCAGGAGCAAGGTGAGATGACAGGAGATCCTGCCCCGGCACTTCG
CCCAATAGCAGCCAGTCCCTTCCCGCTTCAGTGACAACGTCGAGCACAGCTGCGCAAGGAACGCCCGTCGTGGCCAGCCACGATA
GCCGCGCTGCCTCGTCCTGCAGTTCATTCAGGGCACCGGACAGGTCGGTCTTGACAAAAAGAACCGGGCGCCCCTGCGCTGACAG
CCGGAACACGGCGGCATCAGAGCAGCCGATTGTCTGTTGTGCCCAGTCATAGCCGAATAGCCTCTCCACCCAAGCGGCCGGAGAAC
CTGCGTGCAATCCATCTTGTTCAATCATGCGAAACGATCCTCATCCTGTCTCTTGATCAGATCCGAAAATGGATATACAAGCTCCCGG
GAGCTTTTTGCAAAAGCCTAGGCCTCCAAAAAAGCCTCCTCACTACTTCTGGAATAGCTCAGAGGCAGAGGCGGCCTCGGCCTCTG
CATAAATAAAAAAAATTAGTCAGCCATGGGGCGGAGAATGGGCGGAACTGGGCGGAGTTAGGGGCGGGATGGGCGGAGTTAGGGG
CGGGACTATGGTTGCTGACTAATTGAGATGCATGCTTTGCATACTTCTGCCTGCTGGGGAGCCTGGGGACTTTCCACACCTGGTTGC
TGACTAATTGAGATGCATGCTTTGCATACTTCTGCCTGCTGGGGAGCCTGGGGACTTTCCACACCCTAACTGACACACATTCCACAG
AATTAATTCGCGTTAAATTTTTGTTAAATCAGCTCATTTTTTAACCAATAGGCCGAAATCGGCAAAATCCCTTATAAATCAAAAGAATA
GACCGAGATAGGGTTGAGTGTTGTTCCAGTTTGGAACAAGAGTCCACTATTAAAGAACGTGGACTCCAACGTCAAAGGGCGAAAA
ACCGTCTATCAGGGCGATGGCCCACTACGTGAACCATCACCCTAATCAAGTTTTTTGGGGTCGAGGTGCCGTAAAGCACTAAATCGG
AACCCTAAAGGGAGCCCCCGATTTAGAGCTTGACGGGGAAAGCCGGCGAACGTGGCGAGAAAGGAAGGGAAGAAAGCGAAAGG
AGCGGGCGCTAGGGCGCTGGCAAGTGTAGCGGTCACGCTGCGCGTAACCACCACACCCGCCGCGCTTAATGCGCCGCTACAGGGC
GCGTGGGGATACCCCCTAGAGCCCCAGCTGGTTCTTTCCGCCTCAGAAGCCATAGAGCCCACCGCATCCCCAGCATGCCTGCTATTG
TCTTCCCAATCCTCCCCCTTGCTGTCCTGCCCCACCCCACCCCCCAGAATAGAATGACACCTACTCAGACAATGCGATGCAATTTCCT
CATTTTATTAGGAAAGGACAGTGGGAGTGGCACCTTCCAGGGTCAAGGAAGGCACGGGGGAGGGGCAAACAACAGATGGCTGGC
AACTAGAAGGCACAGTCGAGGCTGATCAGCGGGTTTAAACTCAATGGTGATGGTGATGATGACCGGTACGCGTAGAATCGAGACCG
AGGAGAGGGTTAGGGATAGGCTTACCTTCGAAGGGCCCTCTAGAGCTGGTGCTGTTTTCTTCTGCAGGTAAAGCTGGGCTGGATGA
GCCCCCACCTTGCTCTTCTTCCTCCATGGCAGAGTCCTGAGAGCTTGAACCCTTAGCCCCAATTTGGGTCTGGGCCTCCTCACCACC
ATCACCCCCACCACCGCCAACACCACACCCACCACCCTCGCCTCCACCCTCTTCCATGGGCTCCAGCCCCAATCCATCCAGTTCTGA

GAAGAGGGGGTCATCAGAGTGGCCAGGATCTTGAACGCTGCTGCCACAGTCCACACACGCCGTCACATCAAGGGCCCGAGGCAGC
TGGCCCTTCCGGACCCAGGAGTGCACAGCACCCAGTTCCCGCCTCTGGTCCTCTGAGAACCGTGGCTGCTGTTTCTGCATGGCCCG
GAGCCTCTCCCGGTCTTGCTTCAGCACAGAGGCTGCATCCCTGGACGGCACCTGGTATGTCATCATGACACGCTGGTCGGCATTGGC
CATGAGCAGCCAGATGGGGTCCTGGAGCACACACTTAATGACCTGGTCCCCAGGCTCAGTCCTGGCCCGAGCAGCCCCAGCTTCAG
CCTCGGAAGAGTCGATGCTGCCAAAGTACTTGCTTGTATGGCTGCTCTGACTGCTGCGGGTGATGCTGGCTGAGGTGCTTCCCCCTT
CGTGGGATCCTGAACCAGACCCAGAGCCCAGGCCAGAGCCCAGGGAGCCTGAGGCTGCGGAGCCTGTGCCCGAGCGAGAGTCTT
CTTGGAGCAGTAGCTCCAGCAGGTCGCTGGAGCCTGAAAGTGCATCCTGATTGGACGACTCAGTAACCTCCACCAATCTGGCCTCT
GGCTCAGCAGTCTCCTCACTGGGAGGCAGGGGCCCAGCACTGCTTCCTGGGCCTCCTGCAGCAGCGCCCCCCTCCGTGCGGGGGG
ACTCCTCAAGCTGCAGCAGATTGAGCTGGAGTGGGGAGCTGCATCTCGAGTTGAACAGTGGGGAGTCTGGGCGGTGGGGGGGGCT
GAGAGGTGGTGGGGGCAGGGATGGAGAGGGCGAGTGGGAAGCAGGCGTGGGTGGCCCCTCAACAGGGGCCTGGGACACCCCATA
TGGATAACTAGGTGGGGTAGGGAATAGATAGTTAGGGAGCACCAAGGCCACCATTGGGGTCACTAAGGGAGAAGGGAAGGTAGCA
GGGGACACAGATGTAGGGGCAGGGGGAAGGGGCTGGGGTCCTCCTCGAGGGGAGAATACTGGGAGTGGGTAGGGCTGGACCATT
GCTGGGAAGGGGGTCGTGGCTGGTGGGGGTGGCCAGGGTCCAGAAGAGGGCACAGGTGAAGGATGGGAGACATAGCAGGGAGTT
TCGGGCCGGGGGGTCTGGTGGTGGTGGTGGCGGGAACGCTTTGCTTTAGATCGGCAGTGGTGTCGGCGACCAGGGGGAATGGGGC
CATGGTGGCAGCCAGGGGCTGAGGGGGCCACAGAAGAGGTGTCAAGTCCACGAAGCCTGCCAAGAATCTCTGAAGCGGTTGAGG
AAGGCTTGCTCTTCCTTCTGTGTGTGCAGGGACAGCACGGCCTTGGTCAGACCCACTGGGCGATAAGCATCTGGGGTTGGGTCAGG
GGCTACTGTGGGGCTGGGGGCCGGACTGGGGGCTGGGCCAGGGGCCAGGCCAGGCAGGTCTTCCATCATGATGATGTCCGACTCC
GGGGGCTTCTTGTCTCCCACATGGACGATGGTGGAGCTGAAGCTACACTGACTGGTGACGGACACCACGCTCTCTGCCTTATTGGC
CAGGGCGAGCGGGCTCAGGGTGCCTCCCACCACTGGCTCCTTCCGAGGAGTTGCCCCCTCCCCAGACAGCATTGCTGACGACGGA
TTTTTCTTGGCCCCCACAGGAACTGGACCTGCCCTCTGCTTGTCATCATCAGAGGCTGAAGAGGCAGTGTAGGAGGAGGAGGAGG
CACATTTACGCTTGGTTGTACTGGGAATGTTGCAGCTCTCCAAATACCTGAGGATGCTGTCCAGGCAGTTGATCTGCTGGTAGGAAC
AGCCAGAGGTTTCTTTCCTCTCTGGCTCCTCAGGGGCCAAGGCTAACGAGGCTTGGTCAGGAACTGGGGCCACCTCCAGTTCGGG
GTTTGGGGACTGGCAGGGAAGGACTTTGGCTTTGAATGTACCTGTAGCAAGGAGGCGGGGCCGGGGTGGGGGCTTGGCCCGAGAT
TCAATGAAGAGCTGTTGTCCCTGGTGCTTTACCAGATGCACATCCTTACAGATCTGCTGGAAAGTCACTGGAGCAGGAGGCCCAGG
CCCCTCAGCGTCCCCCCCATTGCTATCACTGGAGGAGCCAGGGCTGTGTAGAGGACCAGGGGACATCAGAGGGCCAACTCCACAG
AGCCCCGTGGGGCTGGAGCTGTGCACAGGCTGCAGCAGCAATCGATGGATCTGCTCTGAGAGCTCCTGGATATCAGAGTCCAGGGA
CGGAGCTGGGCTGGGGGCTGGGGGAGTGAAGACGTCCTCATTCAGGGGTGCCGTGCGCACTTTATGGCGACCCAACACGAAAGCC
ACCTTGCGGCTCCAGGGGTGCACAAAACCGGCCCAGCTGGTGTCCATGGTGACATATTCCCCGTTCCGAGCACAGAAGCGAATAGG
GGAATGGTCAAAGGGCTGGCCTGCCAGCTGCAGTATCTTCTTATGAATGGCCAGCATGAGGGGTCGGTCCTCAGGATGTAGAAAGA
GAAGTACTGGAGCCCCCAGGAATCCTGGGGAAGGTAACCCAGCAGTGGGGCAGCCCTTTCATCTACATCCTGGAAGAGGCAGCTT
GGTGTGTGTCGGGTGGTGAAGATCCTCTTGTCAGGAGGGATCCGGGGAGCTTCATAACCAGAGTGGATGCGCTCGGCAATGAGTAG
GCAGCACGGCTGTGCAGGGGCTCCATCTGAGACCCGAATCTTGGTCACATATGGGGTTAGGCGGAATGGCTGGTACCGAGGCCCTG
GATCCCGGTCAGGACCTCCTCTGATTCGGCAGAAGACAGACTTTTCCTGGGTGAAGTCCTTGAGACCTGAACCTGCAGAGGTGCCA
GTGCCCCAGGTGGGCAGTCGAGATGGTGTAGTAGAGCCATAGAAGACACCCACATCCTGGGGAGCCAGGAGCTCTGAGAAGCGGG
CACCCCGAAACACATCCCGTTTGCAACGCAGCAGGACACCTGCCTGCTCCGAAATATAGACAATCCGGCCTGTCAGGAAGGACAC
AGCCACAGAGAAGGTGTCCTGGTTTCGAAGTGTGTATTCGGATGTGATATGCTCCAATTCCTCCAGGGTGTAAGTAGACATGTCCAT
GGCACAAGGCTCACCCTCCTCCAGACTCCACTGCTGGTAATATTCCTGGTTAGCCTGAACCTGCTTGACACAGGCCAGAGCGTACT
GCAGTGTGGCCAAGGTCCCAGAGCGGCCCTTGCCCCGACGCTCTGGTGGCAGTCGAAGTTTGAGCTCCCGAAGTGCAGTCATGAG
TTCTTTCTGGGTCCTGGCTCGAGCTGACTGTTCACTGCTGCAGCCACTGGTAGATGGGTTGTCCTGCTCTGAGCTCGCACTCAGGAG
GCTGTAGGCAATGGAGCTGCTGGGTGGGGATGGGCTCTGTGAGTTTGTACTCTTGCTGCTCTCAGTGGTCTCCAGCAGAGCTGAGT
CCTTGCCATTGCCAGAAGAGGAACTATGAGAACTCCGCTGAGATGCGCCCCTGGACTCGGGTCCGTTGGACTCATTGCCACTTGAG
CCATTGCTGTTTGCATCAGTGTCATCAGCCAGGCTGGGGCCTGGACAAGGCCGGTGCTGCGGGGCCCCAGGGGATGGGACTCCTCC
AGGACAAAAAGGTTCTCCGGGCCTGGGGTCTCCTCCCCCATCGGCCCCTTCTAGGGGACCACTCATTCTAGACTCGAGCGGCCGCC
ACTGTGCTGGATATCTGCAGAATTCCACCACACTGGACTAGTGGATCCGAGCTCGGTACCAAGCTTAACTAGCCAGCTTGGGTCTCC
CTATAGTGAGTCGTATTAATTTCGATAAGCCAGTAAGC

C, pGST-*hPer1* map

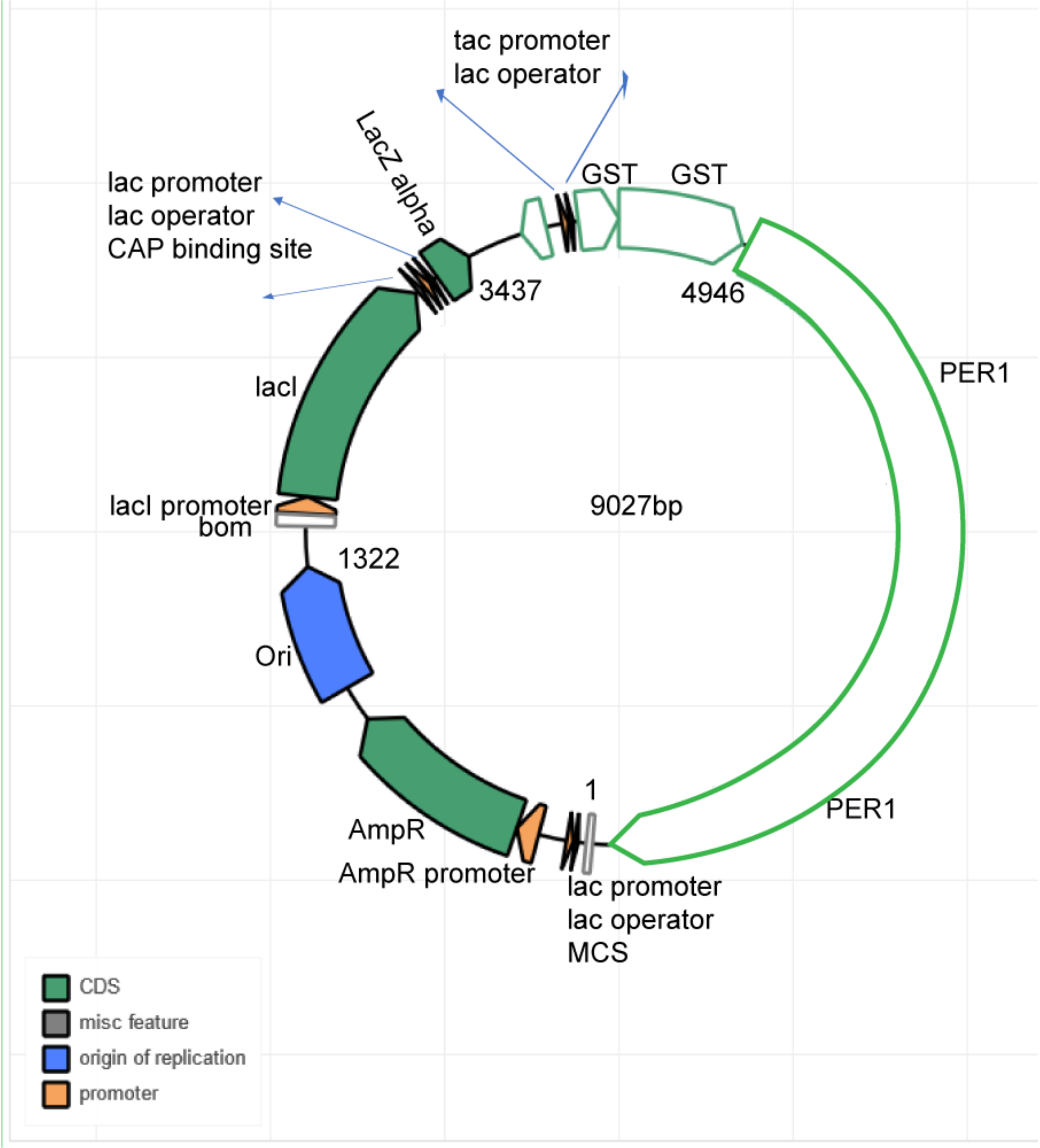

D, pGST-*hPer1* sequence

>pGSThPer1
ATTGTTATCCGCTCACAATTCCACACAACATACGAGCCGGAAGCATAAAGTGTAAAGCCTGGGGTGTAGCGATAGCGGAGTAATTCT
TGAAGACGAAAGGGCCTCGTGATACGCCTATTTTTATAGGTTAATGTCATGATAATAATGGTTTCTTAGACGTCAGGTGGCACTTTTC
GGGGAAATGTGCGCGGAACCCCTATTTGTTTATTTTTCTAAATACATTCAAATATGTATCCGCTCATGAGACAATAACCCTGATAAATG
CTTCAATAATATTGAAAAAGGAAGAGTATGAGTATTCAACATTTCCGTGTCGCCCTTATTCCCTTTTTTGCGGCATTTTGCCTTCCTGT
TTTTGCTCACCCAGAAACGCTGGTGAAAGTAAAAGATGCTGAAGATCAGTTGGGTGCACGAGTGGGTTACATCGAACTGGATCTCA
ACAGCGGTAAGATCCTTGAGAGTTTTCGCCCCGAAGAACGTTTTCCAATGATGAGCACTTTTAAAGTTCTGCTATGTGGCGCGGTAT
TATCCCGTGTTGACGCCGGGCAAGAGCAACTCGGTCGCCGCATACACTATTCTCAGAATGACTTGGTTGAGTACTCACCAGTCACAG
AAAAGCATCTTACGGATGGCATGACAGTAAGAGAATTATGCAGTGCTGCCATAACCATGAGTGATAACACTGCGGCCAACTTACTTC
TGACAACGATCGGAGGACCGAAGGAGCTAACCGCTTTTTTGCACAACATGGGGGATCATGTAACTCGCCTTGATCGTTGGGAACCG
GAGCTGAATGAAGCCATACCAAACGACGAGCGTGACACCACGATGCCTGCAGCAATGGCAACAACGTTGCGCAAACTATTAACTG
GCGAACTACTTACTCTAGCTTCCCGGCAACAATTAATAGACTGGATGGAGGCGGATAAAGTTGCAGGACCACTTCTGCGCTCGGCCC
TTCCGGCTGGCTGGTTTATTGCTGATAAATCTGGAGCCGGTGAGCGTGGGTCTCGCGGTATCATTGCAGCACTGGGGCCAGATGGTA
AGCCCTCCCGTATCGTAGTTATCTACACGACGGGGAGTCAGGCAACTATGGATGAACGAAATAGACAGATCGCTGAGATAGGTGCCT
CACTGATTAAGCATTGGTAACTGTCAGACCAAGTTTACTCATATATACTTTAGATTGATTTAAAACTTCATTTTTAATTTAAAAGGATCT
AGGTGAAGATCCTTTTTGATAATCTCATGACCAAAATCCCTTAACGTGAGTTTTCGTTCCACTGAGCGTCAGACCCCGTAGAAAAGA
TCAAAGGATCTTCTTGAGATCCTTTTTTTCTGCGCGTAATCTGCTGCTTGCAAACAAAAAAACCACCGCTACCAGCGGTGGTTTGTT
TGCCGGATCAAGAGCTACCAACTCTTTTTCCGAAGGTAACTGGCTTCAGCAGAGCGCAGATACCAAATACTGTCCTTCTAGTGTAGC
CGTAGTTAGGCCACCACTTCAAGAACTCTGTAGCACCGCCTACATACCTCGCTCTGCTAATCCTGTTACCAGTGGCTGCTGCCAGTG
GCGATAAGTCGTGTCTTACCGGGTTGGACTCAAGACGATAGTTACCGGATAAGGCGCAGCGGTCGGGCTGAACGGGGGGTTCGTGC
ACACAGCCCAGCTTGGAGCGAACGACCTACACCGAACTGAGATACCTACAGCGTGAGCTATGAGAAAGCGCCACGCTTCCCGAAG
GGAGAAAGGCGGACAGGTATCCGGTAAGCGGCAGGGTCGGAACAGGAGAGCGCACGAGGGAGCTTCCAGGGGGAAACGCCTGG
TATCTTTATAGTCCTGTCGGGTTTCGCCACCTCTGACTTGAGCGTCGATTTTTGTGATGCTCGTCAGGGGGGCGGAGCCTATGGAAA
AACGCCAGCAACGCGGCCTTTTTACGGTTCCTGGCCTTTTGCTGGCCTTTTGCTCACATGTTCTTTCCTGCGTTATCCCCTGATTCTG
TGGATAACCGTATTACCGCCTTTGAGTGAGCTGATACCGCTCGCCGCAGCCGAACGACCGAGCGCAGCGAGTCAGTGAGCGAGGA
AGCGGAAGAGCGCCTGATGCGGTATTTTCTCCTTACGCATCTGTGCGGTATTTCACACCGCATAAATTCCGACACCATCGAATGGCG
CAAAACCTTTCGCGGTATGGCATGATAGCGCCCGGAAGAGAGTCAATTCAGGGTGGTGAATGTGAAACCAGTAACGTTATACGATG
TCGCAGAGTATGCCGGTGTCTCTTATCAGACCGTTTCCCGCGTGGTGAACCAGGCCAGCCACGTTTCTGCGAAAACGCGGGAAAAA
GTGGAAGCGGCGATGGCGGAGCTGAATTACATTCCCAACCGCGTGGCACAACAACTGGCGGGCAAACAGTCGTTGCTGATTGGCG
TTGCCACCTCCAGTCTGGCCCTGCACGCGCCGTCGCAAATTGTCGCGGCGATTAAATCTCGCGCCGATCAACTGGGTGCCAGCGTG
GTGGTGTCGATGGTAGAACGAAGCGGCGTCGAAGCCTGTAAAGCGGCGGTGCACAATCTTCTCGCGCAACGCGTCAGTGGGCTGA
TCATTAACTATCCGCTGGATGACCAGGATGCCATTGCTGTGGAAGCTGCCTGCACTAATGTTCCGGCGTTATTTCTTGATGTCTCTGA
CCAGACACCCATCAACAGTATTATTTTCTCCCATGAAGACGGTACGCGACTGGGCGTGGAGCATCTGGTCGCATTGGGTCACCAGCA
AATCGCGCTGTTAGCGGGCCCATTAAGTTCTGTCTCGGCGCGTCTGCGTCTGGCTGGCTGGCATAAATATCTCACTCGCAATCAAATT
CAGCCGATAGCGGAACGGGAAGGCGACTGGAGTGCCATGTCCGGTTTTCAACAAACCATGCAAATGCTGAATGAGGGCATCGTTCC
CACTGCGATGCTGGTTGCCAACGATCAGATGGCGCTGGGCGCAATGCGCGCCATTACCGAGTCCGGGCTGCGCGTTGGTGCGGATA
TCTCGGTAGTGGGATACGACGATACCGAAGACAGCTCATGTTATATCCCGCCGTTAACCACCATCAAACAGGATTTTCGCCTGCTGG
GGCAAACCAGCGTGGACCGCTTGCTGCAACTCTCTCAGGGCCAGGCGGTGAAGGGCAATCAGCTGTTGCCCGTCTCACTGGTGAA
AAGAAAAACCACCCTGGCGCCCAATACGCAAACCGCCTCTCCCCGCGCGTTGGCCGATTCATTAATGCAGCTGGCACGACAGGTTT
CCCGACTGGAAAGCGGGCAGTGAGCGCAACGCAATTAATGTAAGTTAGCTCACTCATTAGGCACCCCAGGCTTTACACTTTATGCTT
CCGGCTCGTATGTTGTGTGGAATTGTGAGCGGATAACAATTTCACACAGGAAACAGCTATGACCATGATTACGGATTCACTGGCCGT
CGTTTTACAACGTCGTGACTGGGAAAACCCTGGCGTTACCCAACTTAATCGCCTTGCAGCACATCCCCCTTTCGCCAGCTGGCGTAA
TAGCGAAGAGGCCCGCACCGATCGCCCTTCCCAACAGTTGCGCAGCCTGAATGGCGAATGGCGCTTTGCCTGGTTTCCGGCACCAG
AAGCGGTGCCGGAAAGCTGGCTGGAGTGCGATCTTCCTGAGGCCGATACTGTCGTCGTCCCCTCAAACTGGCAGATGCACGGTTAC
GATGCGCCCATCTACACCAACGTGACCTATCCCATTACGGTCAATCCGCCGTTTGTTCCCACGGAGAATCCGACGGGTTGTTACTCG
CTCACATTTAATGTTGATGAAAGCTGGCTACAGGAAGGCCAGACGCGAATTATTTTTGATGGCGTTGGAATTAGCTTATCGACTGCAC
GGTGCACCAATGCTTCTGGCGTCAGGCAGCCATCGGAAGCTGTGGTATGGCTGTGCAGGTCGTAAATCACTGCATAATTCGTGTCGC
TCAAGGCGCACTCCCGTTCTGGATAATGTTTTTTGCGCCGACATCATAACGGTTCTGGCAAATATTCTGAAATGAGCTGTTGACAATT
AATCATCGGCTCGTATAATGTGTGGAATTGTGAGCGGATAACAATTTCACACAGGAAACAGTATTCATGTCCCCTATACTAGGTTATT
GGAAAATTAAGGGCCTTGTGCAACCCACTCGACTTCTTTTGGAATATCTTGAAGAAAAATATGAAGAGCATTTGTATGAGCGCGATG
AAGGTGATAAATGGCGAAACAAAAAGTTTGAATTGGGTTTGGAGTTTCCCAATCTTCCTTATTATATTGATGGTGATGGAAGAAAAA
TATGAAGAGCATTTGTATGAGCGCGATGAAGGTGATAAATGGCGAAACAAAAAGTTTGAATTGGGTTTGGAGTTTCCCAATCTTCCT
TATTATATTGATGGTGATGTTAAATTAACACAGTCTATGGCCATCATACGTTATATAGCTGACAAGCACAACATGTTGGGTGGTTGTCC
AAAAGAGCGTGCAGAGATTTCAATGCTTGAAGGAGCGGTTTTGGATATTAGATACGGTGTTTCGAGAATTGCATATAGTAAAGACTT
TGAAACTCTCAAAGTTGATTTTCTTAGCAAGCTACCTGAAATGCTGAAAATGTTCGAAGATCGTTTATGTCATAAAACATATTTAAAT
GGTGATCATGTAACCCATCCTGACTTCATGTTGTATGACGCTCTTGATGTTGTTTTATACATGGACCCAATGTGCCTGGATGCGTTCCC
AAAATTAGTTTGTTTTAAAAAACGTATTGAAGCTATCCCACAAATTGATAAGTACTTGAAATCCAGCAAGTATATAGCATGGCCTTTG
CAGGGCTGGCAAGCCACGTTTGGTGGTGGCGACCATCCTCCAAAATCGGATCTGATCGAAGGTCGTGGGATCCCCAACCATATGAG
TGGCCCCCTAGAAGGGGCTGATGGGGGAGGGGACCCCAGGCCTGGGGAATCATTTTGTCCTGGGGGCGTCCCATCCCCTGGGCCCC
CACAGCACCGGCCTTGCCCAGGCCCCAGCCTGGCCGATGACACCGATGCCAACAGCAATGGTTCAAGTGGCAATGAGTCCAACGG
GCATGAGTCTAGAGGCGCATCTCAGCGGAGCTCACACAGCTCCTCCTCAGGCAACGGCAAGGACTCAGCCCTGCTGGAGACCACT
GAGAGCAGCAAGAGCACAAACTCTCAGAGCCCATCCCCACCCAGCAGTTCCATTGCCTACAGCCTCCTGAGTGCCAGCTCAGAGC
AGGACAACCCGTCCACCAGTGGCTGCAGCAGTGAACAGTCAGCCCGGGCAAGGACTCAGAAGGAACTCATGACAGCACTTCGAG
AGCTCAAGCTTCGACTGCCGCCAGAGCGCCGGGGCAAGGGCCGCTCTGGGACCCTGGCCACGCTGCAGTACGCACTGGCCTGTGT
CAAGCAGGTGCAGGCCAACCAGGAATACTACCAGCAGTGGAGCCTGGAGGAGGGCGAGCCTTGCTCCATGGACATGTCCACCTAT
ACCCTGGAGGAGCTGGAGCACATCACGTCTGAGTACACACTTCAGAACCAGGATACCTTCTCAGTGGCTGTCTCCTTCCTGACGGG
CCGAATCGTCTACATTTCGGAGCAGGCAGCCGTCCTGCTGCGTTGCAAGCGGGACGTGTTCCGGGGTACCCGCTTCTCTGAGCTCC
TGGCTCCCCAGGATGTGGGAGTCTTCTATGGTTCCACTGCTCCATCTCGCCTGCCCACCTGGGGCACAGGGGCCTCAGCAGGTTCA

GGCCTCAGGGACTTTACCCAGGAGAAGTCCGTCTTCTGCCGTATCAGAGGAGGTCCTGACCGGGATCCAGGGCCTCGGTACCAGCC
ATTCCGCCTAACCCCGTATGTGACCAAGATCCGGGTCTCAGATGGGGCCCCTGCACAGCCGTGCTGCCTGCTGATTGCAGAGCGCAT
CCATTCGGGTTACGAAGCTCCCCGGATACCCCCTGACAAGAGGATTTTCACTACGCGGCACACACCCAGCTGCCTCTTCCAGGATGT
GGATGAAAGGGCTGCCCCCCTGCTGGGCTACCTGCCCCAGGACCTCCTGGGGGCCCCAGTGCTCCTGTTCCTGCATCCTGAGGACC
GACCCCTCATGCTGGCTATCCACAAGAAGATTCTGCAGTTGGCGGGCCAGCCCTTTGACCACTCCCCTATCCGCTTCTGTGCCCGCA
ACGGGGAGTATGTCACCATGGACACCAGCTGGGCTGGCTTTGTGCACCCCTGGAGCCGCAAGGTAGCCTTCGTGTTGGGCCGCCAC
AAAGTACGCACGGCCCCCCTGAATGAGGACGTGTTCACTCCCCCGGCCCCCAGCCCAGCTCCCTCCCTGGACACTGATATCCAGGA
GCTGTCAGAGCAGATCCACCGGCTGCTGCTGCAGCCCGTCCACAGCCCCAGCCCCACGGGACTCTGTGGAGTCGGCGCCGTGACA
TCCCCAGGCCCTCTCCACAGCCCTGGGTCCTCCAGTGATAGCAACGGGGGTGATGCAGAGGGGCCTGGGCCTCCTGCGCCAGTGA
CTTTCCAGCAGATCTGTAAGGATGTGCATCTGGTGAAGCACCAGGGCCAGCAGCTTTTTATTGAGTCTCGGGCCCGGCCTCAGTCCC
GGCCCCGCCTCCCTGCTACAGGCACGTTCAAGGCCAAGGCCCTTCCCTGCCAATCCCCAGACCCAGAGCTGGAGGCGGGTTCTGCT
CCCGTCCAGGCCCCACTAGCCTTGGTCCCTGAGGAGGCCGAGAGGAAAGAAGCCTCCAGCTGCTCCTACCAGCAGATCAACTGCC
TGGACAGCATCCTCAGGTACCTGGAGAGCTGCAACCTCCCCAGCACCACTAAGCGTAAATGTGCCTCCTCCTCCTCCTATACCACCT
CCTCAGCCTCTGACGACGACAGGCAGAGGACAGGTCCAGTCTCTGTGGGGACCAAGAAGATCCGCCGTCAGCAGCGCTGTCTGGG
GAGGGGGCCACCCCACGGAAGGAGCCAGTGGTGGGAGGCACCCTGAGCCCGCTCGCCCTGGCCAATAAGGCGGAGAGTGTGGTG
TCCGTCACCAGTCAGTGTAGCTTCAGCTCCACCATCGTCCATGTGGGAGACAAGAAGCCCCCGGAGTCGGACATCATCATGATGGA
GGACCTGCCTGGTCTAGCCCCAGGCCCAGCCCCCAGCCCAGCCCCCAGCCCCACAGTAGCCCCTGACCCAGCCCCAGACGCCTAC
CGTCCAGTGGGGCTGACCAAGGCCGTGCTGTCCCTGCACACGCAGAAGGAAGAGCAAGCCTTCCTCAGCCGCTTCCGAGACCTGG
GCAGGCTGCGTGGACTCGACAGCTCTTCCACAGCTCCCTCAGCCCTTGGCGAGCGAGGCTGCCACCACGGCCCCGCACCCCCAAG
CCGCCGACACCACTGCCGATCCAAAGCCAAGCGCTCACGCCACCACCAGAACCCTCGGGCTGAAGCGCCCTGCTATGTCTCACAC
CCCTCACCCGTGCCACCCTCCACCCCCTGGCCCACCCCACCAGCCACTACCCCCTTCCCAGCGGTTGTCCAGCCCTACCCTCTCCCA
GTGTTCTCTCCTCGAGGAGGCCCCCAGCCTCTTCCCCCTGCTCCCACATCTGTGCCCCCAGCTGCTTTCCCCGCCCCTTTGGTGACC
CCAATGGTGGCCTTGGTGCTCCCTAACTATCTGTTCCCAACCCCATCCAGCTATCCTTATGGGGCACTCCAGACCCCTGCTGAAGGG
CCTCCCACTCCTGCCTCGCACTCCCCTTCTCCATCCTTGCCCGCCCTCCCCCCGAGTCCTCCTCACCGCCCGGACTCTCCACTGTTCA
ACTCGAGATGCAGCTCTCCACTCCAGCTCAATCTGCTGCAGCTGGAGGAGCTCCCCCGTGCTGAGGGGGCTGCTGTTGCAGGAGG
CCCTGGGAGCAGTGCCGGGCCCCCACCTCCCAGTGCGGAGGCTGCTGAGCCAGAGGCCAGACTGGCGGAGGTCACTGAGTCCTCC
AATCAGGACGCACTTTCCGGCTCCAGTGACCTGCTCGAACTTCTGCTGCAAGAGGACTCGCGCTCCGGCACAGGCTCCGCAGCCTC
GGGCTCCTTGGGCTCTGGCTTGGGCTCTGGGTCTGGTTCAGGCTCCCATGAAGGGGGCAGCACCTCAGCCAGCATCACTCGCAGCA
GCCAGAGCAGCCACACAAGCAAATACTTTGGCAGCATCGACTCTTCCGAGGCTGAGGCTGGGGCTGCTCGGGGCGGGGCTGAGCC
TGGGGACCAGGTGATTAAGTACGTGCTCCAGGATCCCATTTGGCTGCTCATGGCCAATGCTGACCAGCGCGTCATGATGACCTACCA
GGTGCCCTCCAGGGACATGACCTCTGTGCTGAAGCAGGATTGGGAGCGGCTCCGAGCCATGCAGAAGCAGCAGCCTCGGTTTTCT
GAGGACCAGCGGCGGGAACTGGGTGCTGTGCACTCCTGGGTCCGGAAGGGCCAACTGCCTCGGGCTCTTGATGTGATGGCCTGTG
TGGACTGTGGGAGCAGCACCCAGATCCTGGTCACCCTGATGACCCACTCTTCTCAGAGCTGGATGGACTGGGGCTGGAGCCCATGG
AAGAGGGTGGAGGCGAGCAGGGCAGCAGCGGTGGCGGCAGTGGTGAGGGAGAGGGCTGCGAGGAGGCCCAAGGCGGGGCCAA
GGCTTCAAGCTCTCAGGACTTGGCTATGGAGGAGGAGGAAGAAGGCAGGAGCTCATCCAGTCCAGCCTTACCTACAGCAGGAAAC
TGCACCAGCTAGACTCCATTCTGGGACCATCTCCAGGAGTCCATGAGAGGCTTTCTTCTCCTATGTCCCAATTCTCAGAACTCAGAT
GTGGCTAGACCAACCAGTGGGAAACTGCCCCAGCTTCTCCCACCATAGGGGGCCGGACCCCCATCACCAGCCTAGAGTCGACCTGC
AGGCATGCAAGCTTGGCGTAATCATGGTCATAGCTGTTTCCTGTGTGAA

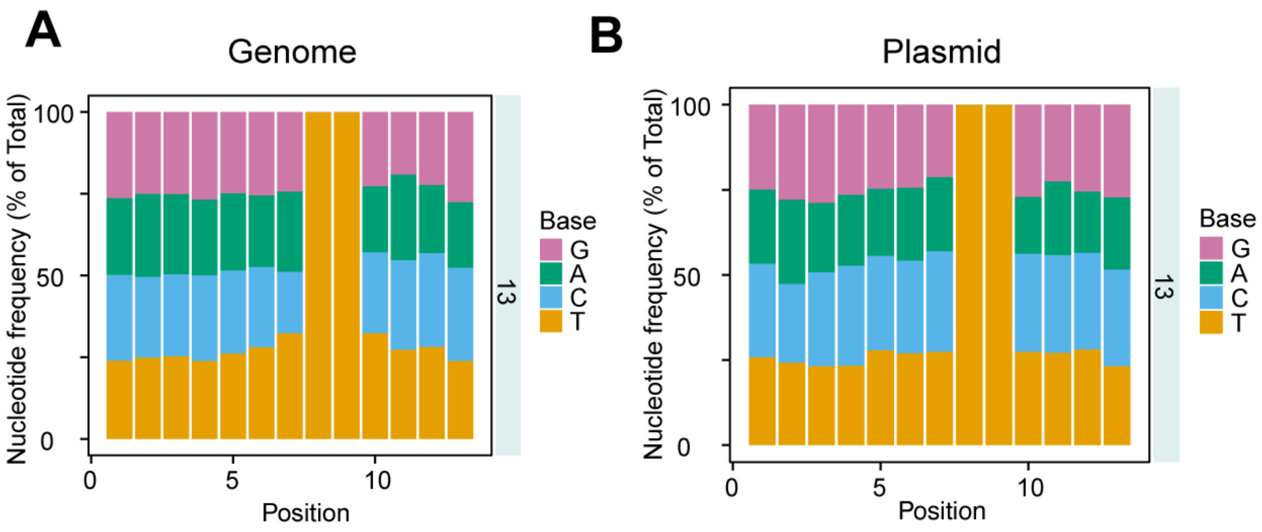

Figure S2 A residues are not present at high frequency immediately 5' to TT residues. All TT dinucleotide-containing 13nt sequences in the *E. coli* genome (A) or plasmid pcDNA-3-*mPer1* were plotted as nucleotide frequency distributions with the TT residues located at positions 8-9 of a 13-nt sequence. The percentage of A residues 5' to the TT is 25% (genome) and 22% (plasmid).

		TS/NTS	
		CHO-AA8 R1	CHO-AA8 R2
bla	rNTP +	1.12	1.12
	rNTP -	1.00	1.03
mPer1	rNTP +	0.66	0.65
	rNTP -	0.63	0.66

Table S1

XR-seq analysis of plasmid repair by CHO-AA8 extracts.

Regions of the plasmid containing the *bla* and *mPer1* genes were analyzed. Repair reactions were conducted with ATP and with or without CTP, GTP and UTP (+/-rNTPs). The extracts do not efficiently transcribe, and no transcription-stimulated repair is evident. Values are given for the ratio of transcribed/non-transcribed strand (TS/NTS) repair in repeat 1 (R1) and repeat 2 (R2).

	Repair Time	TS/NTS		TCR
		mfd^+	mfd^-	(mfd^+/mfd^-)
bla	5min	1.63	0.66	2.47
	12min	1.52	0.64	2.38
	25min	1.43	0.67	2.13
mPer1	5min	0.84	0.77	1.09
	12min	0.80	0.80	1
	25min	0.88	0.85	1.04

Table S2

Transcription-coupled repair in vitro and in vivo by *E. coli* CFE for three DNA repair times.

Additional time points showing transcription-coupled repair in *bla* by *E. coli* extracts in vitro. The 5 min point results are shown in Table1. The 12 and 25 minute time points are shown here.
